# Cardiovascular risk factors, living and ageing in Halle: the CARLA study

**DOI:** 10.1007/s10654-021-00824-7

**Published:** 2022-01-03

**Authors:** Lamiaa Hassan, Ljupcho Efremov, Anne Großkopf, Nadja Kartschmit, Daniel Medenwald, Artjom Schott, Andrea Schmidt-Pokrzywniak, Maria E. Lacruz, Daniel Tiller, Frank Bernhard Kraus, Karin H. Greiser, Johannes Haerting, Karl Werdan, Daniel Sedding, Andreas Simm, Sebastian Nuding, Alexander Kluttig, Rafael Mikolajczyk

**Affiliations:** 1 Institute of Medical Epidemiology, Biostatistics, and Informatics, Medical Faculty of the Martin-Luther-University Halle-Wittenberg, Halle (Saale), Germany; 2 University Clinic and Outpatient Clinic for Cardiac Surgery, Middle German Heart Centre at the University Hospital Halle, Halle, Germany; 3 Department of Radiation Oncology, University Hospital Halle (Saale), Halle (Saale), Germany; 4 Department of Internal Medicine III, University Hospital, Martin-Luther-University Halle-Wittenberg, Halle (Saale), Germany; 5 Central Laboratory, University Hospital Halle, Halle (Saale), Germany; 6 Division of Cancer Epidemiology, German Cancer Research Center, Heidelberg, Germany; 7 Interdisciplinary Center for Health Sciences, Medical Faculty of the Martin-Luther-University Halle-Wittenberg, Halle (Saale), Germany; 8 Clinical Computing Center – Data Integration Center, University Hospital Martin-Luther-University Halle-Wittenberg, Halle (Saale), Germany

**Keywords:** Population-based cohort study, Elderly population, Cardiovascular diseases, Healthy ageing

## Abstract

The CARLA study (Cardiovascular Disease, Living and Ageing in Halle) is a longitudinal population-based cohort study of the general population of the city of Halle (Saale), Germany. The primary aim of the cohort was to investigate risk factors for cardiovascular diseases based on comprehensive cardiological phenotyping of study participants and was extended to study factors associated with healthy ageing. In total, 1779 probands (812 women and 967 men, aged 45–83 years) were examined at baseline (2002–2005), with a first and second follow-up performed 4 and 8 years later. The response proportion at baseline was 64.1% and the reparticipation proportion for the first and second follow-up was 86% and 77% respectively. Sixty-four percent of the study participants were in retirement while 25% were full- or partially-employed and 11% were unemployed at the time of the baseline examination. The currently running third follow-up focuses on the assessment of physical and mental health, with an intensive 4 h examination program, including measurement of cardiovascular, neurocognitive, balance and gait parameters. The data collected in the CARLA Study resulted in answering various research questions in over 80 publications, of which two thirds were pooled analyses with other similar population-based studies. Due to the extensiveness of information on risk factors, subclinical conditions and evident diseases, the biobanking concept for the biosamples, the cohort representativeness of an elderly population, and the high level of quality assurance, the CARLA cohort offers a unique platform for further research on important indicators for healthy ageing.

**Supplementary Information:**

The online version contains supplementary material available at 10.1007/s10654-021-00824-7.

## Background

Cardiovascular diseases (CVDs) have long been considered the leading cause of death in Germany accounting for 37.4% of total deaths in 2018 [1]. In 2000, 10 years after the reunification of Germany, mortality from cardiovascular disease was still about 50% higher in East compared with West Germany [2]. Compared with other federal states in both former West and East Germany, Saxony-Anhalt has the highest cardiovascular disease morbidity and mortality from reunification until today [3]. The population of Saxony-Anhalt is in average older and shows higher rates of cardiovascular risk factors than those of the other German federal states [4]. In a study investigating regional differences in Germany with respect to life-time prevalence of major cardiovascular disease, Saxony-Anhalt was the most unfavorable state [5]. The Cardiovascular Risk Factors, Living and Ageing in Halle (CARLA) Study, a longitudinal population-based cohort study, was designed to address this gap by collecting detailed data on CVD risk factors and follow-up information on CVD morbidity and mortality in a region of eastern Germany characterized by a particularly pronounced CVD mortality. After the baseline examination, two additional follow-up examinations were performed, with a third follow-up currently taking place. Over time, the CARLA study evolved toward a broader research platform on ageing. Understanding the lifecourse trajectories pursued by an elderly population while maintaining an acceptable level of well-being and functionality could establish a useful route to recognize and plan good preventive approaches [6]. The aim of this article is to provide an overview of the CARLA study, and the main results obtained thus far. Additionally, we provide details on the current third follow-up examination of study participants.

## Study design and methodology

### Study population and recruitment

**Fig. 1:**
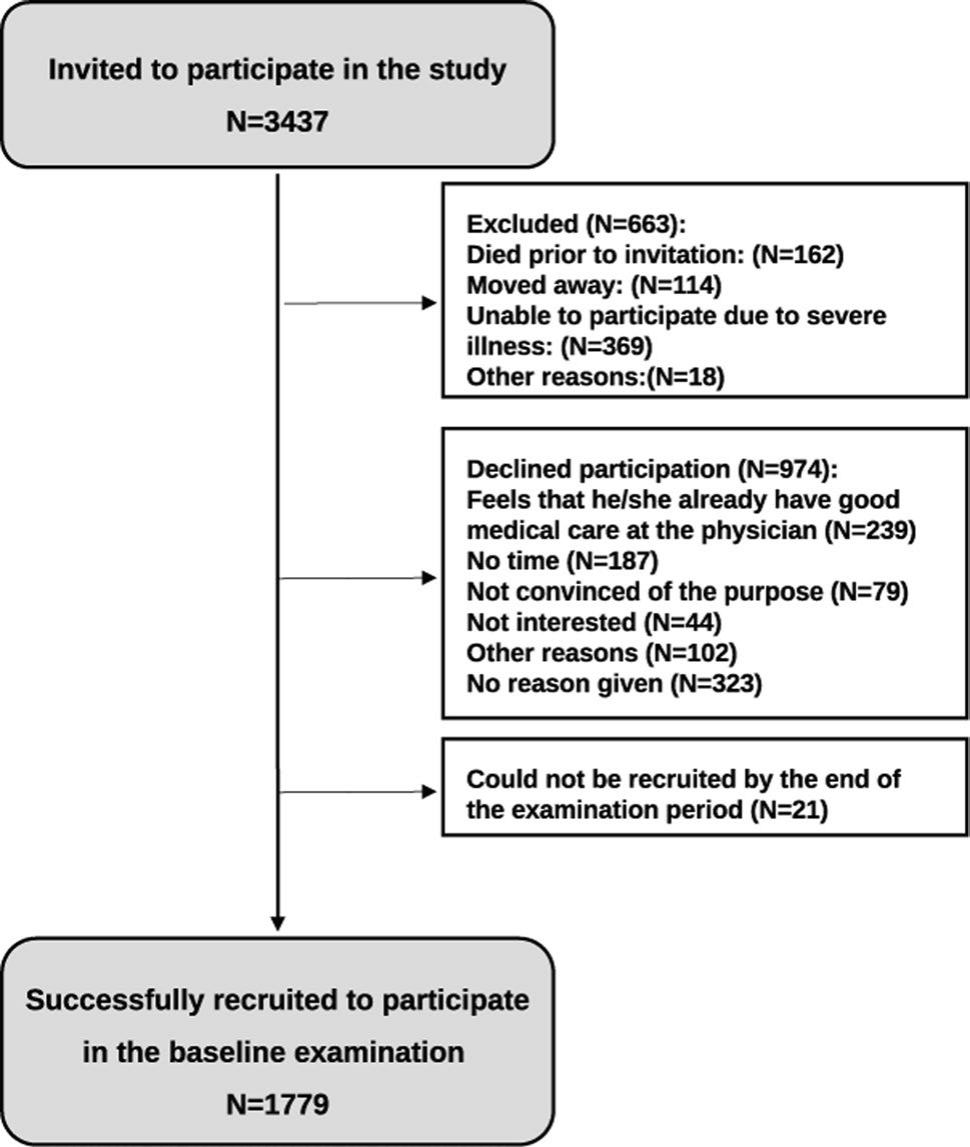
Flowchart of the number of subjects invited for participation in the CARLA study and reasons for exclusion and non-participation

**Fig. 2:**
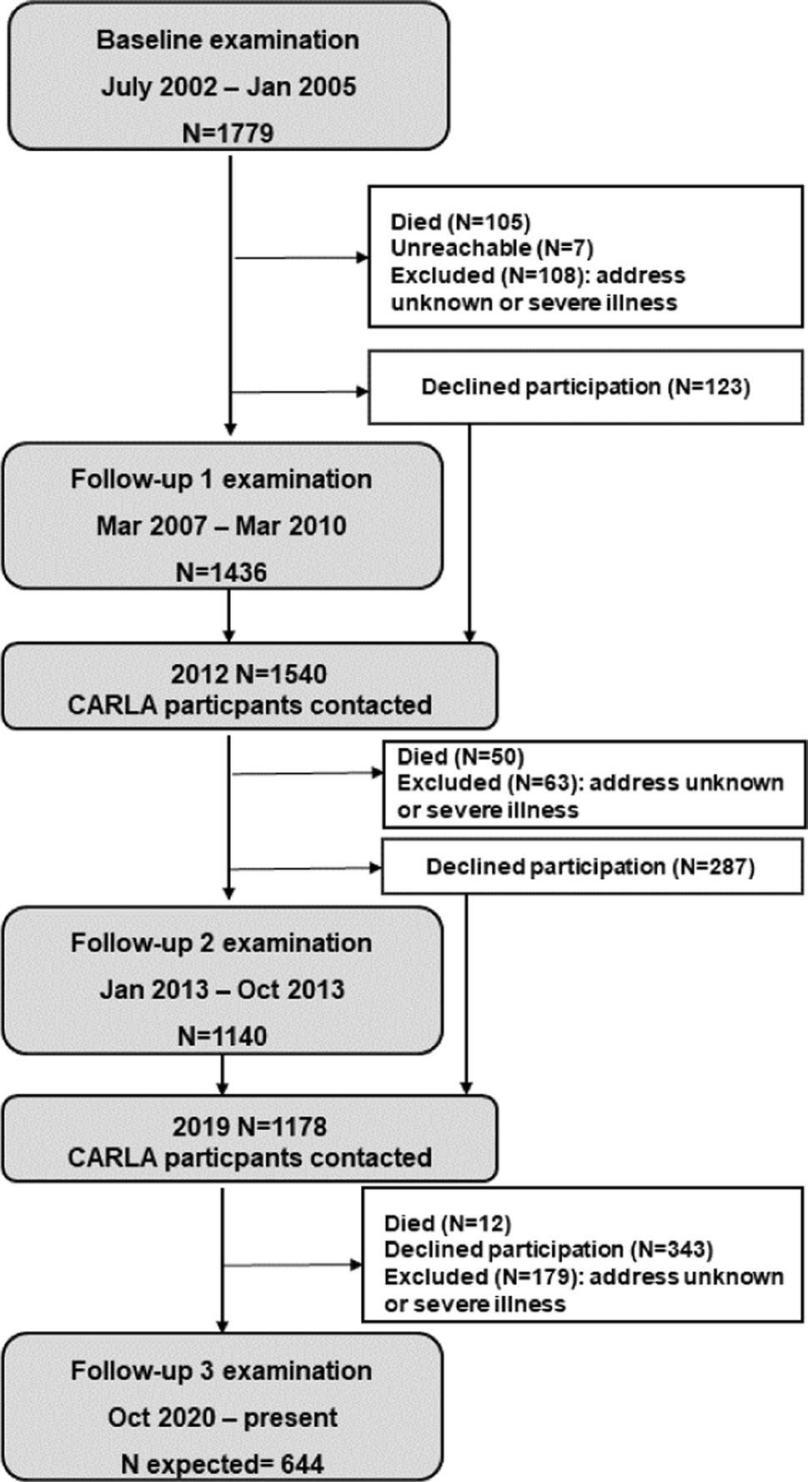
Flowchart of the number of subjects attending examinations for the CARLA cohort and reasons for attrition

A total sample size of 1760 subjects was needed according to the sample size calculation for the study as per the primary outcome "Occurrence of reduced heart rate variability (RHRV)". Details of the sample size calculation have been described elsewhere [7]. A random sample of 2500 men and women each, with an age between 45 and 83 years was drawn from the population registry of the city of Halle (Saale), Saxony-Anhalt in 2002. The detailed recruitment procedure and response rate has been described elsewhere [7, 8]. In brief, the sampling was done using 5-year age strata, where residents in the age group of 75–80 years were oversampled, with twice as many invited from this stratum in comparison to the younger strata. The recruitment of study subjects has been done by inviting consecutive waves of random sub-samples of the original population sample. Accordingly, not all persons originally drawn from the population registry had to be invited in order to achieve a representative sample of the Halle population aged 45–80 years. Of the 3437 subjects invited to participate in the study, 1779 participants were recruited, of which 812 (46%) were women and 967 (54%) men, resulting in a final response proportion of 64.1% (68.6% for men and 59.5% for women) after exclusion of persons who deceased prior to the invitation, moved away or were unable to participate due to illness. The recuirtment process and reasons for non-participation in the study are presented in Fig. 1 and the study flowchart is presented in Fig. 2. Moreover, an analysis of non-respondents was implemented as a means to estimate non-response bias by obtaining information about prevalent diseases and selected behavioral and sociodemographic factors. Compared to the general population in Halle in the age of 45–85 years, CARLA study population had a higher proportion of subjects with university level education (11.8% in the general population in East Germany (2002) vs. 27.7% in CARLA-0) and also a higher proportion of unemployed subjects (for the age group 45–49 years old: 13,47% in the general population of Halle (2006) vs. 27% in the same age group in CARLA-0) [9, 10].

The local ethics commission of the Medical Faculty of the Martin Luther University Halle-Wittenberg approved the study and the follow-up examinations. All participants gave their written informed consent.

### Baseline characteristics of the study population

Analyses of baseline data revealed that the study population suffered from high proportions of cardiovascular risk factors and diseases (Table 1). The study participants were characterized by a very high baseline prevalence of hypertension (74%), type 2 diabetes (15%) and a high average Body Mass Index (BMI) (28 kg/m^2^) [11]. In addition, there was a high prevalence of left ventricular hypertrophy and heart failure (HF) with preserved or reduced ejection fraction (HFpEF and HFrEF). The overall prevalence of symptomatic HF was 9.4% for men and 9.9% for women. Symptomatic HFrEF could be shown in 48% (n = 78), symptomatic HFpEF in 52% (n = 85) of subjects [12].

**Table 1:** Demographic, clinical and laboratory characteristics of the CARLA cohort at baseline (CARLA-0), first (CARLA-1) and second (CARLA-2) follow-up examination

Characteristics	Baseline (CARLA-0)		First Follow-up (CARLA-1)		Second Follow-up (CARLA-2)	
	n	Mean ± SD or n (%)	n	Mean ± SD or n (%)	n	Mean ± SD or n (%)
Age (years)	1779	64.4 ± 10.1	1436	67.3 ± 9.7	1140	70.2 ± 9.2
Female	1779	812 (45.6)	1436	646 (44.9)	1140	529 (46.4)
BMI (kg/m^2^)	1779	28.3 ± 4.7	1423	28.3 ± 4.7	1040	28.9 ± 4.7
Waist circumference (cm)	1779	99.8 ± 12.7	1420	99.5 ± 13.6	0	N/A
SBP (mmHg)	1778	143.9 ± 21.3	1426	137.1 ± 19.6	1044	127.2 ± 18.4
DBP (mmHg)	1778	84.7 ± 11.1	1426	79.9 ± 10.2	1044	73.6 ± 10.4
Triglycerides (mmol/L)	1767	1.89 ± 1.5	1417	1.9 ± 1.2	N/A	N/A
Total cholesterol (mmol/L)	1767	5.5 ± 1.08	1417	5.4 ± 1.05	N/A	N/A
HDL cholesterol (mmol/L)	1767	1.4 ± 0.41	1417	1.3 ± 0.38	N/A	N/A
LDL cholesterol (mmol/L)	1767	3.4 ± 0.89	1416	3.3 ± 0.90	N/A	N/A
Glucose (mmol/L)	1767	5.99 ± 1.83	1417	5.8 ± 1.7	N/A	N/A
HbA1c (%)	1767	5.7 ± 0.8	1417	5.8 ± 0.7	N/A	N/A
Creatinine (μmol/L)	1767	75.2 ± 28.3	1417	80.4 ± 33.8	N/A	N/A
eGFR (mL/min/1.73m^2^)	1767	85.3 ± 16.6	1417	80.1 ± 17.9	N/A	N/A
NT-proBNP (pg/mL)	1722	216.1 ± 580.3	1416	253.3 ± 760.2	N/A	N/A
CRP (mg/L)	1695	3.2 ± 5.9	1417	3.5 ± 7.67	N/A	N/A
IL-6 (pg/mL)	1763	3.5 ± 4.6	1417	3.8 ± 7.4	N/A	N/A
sTNF-R1 (pg/mL)	1764	1180.3 ± 408.8	1417	1317.6 ± 509.4	N/A	N/A
Current smoker	1778	344 (19.3)	1427	224 (15.7)	1134	163 (14.3)
Current sport activity	1777	643 (36.2)	1426	667 (46.7)	1106	540 (48.8)
Average years of education	1779	14.9 ± 2.5	1431	14.8 ± 2.5	N/A	N/A
University education	1778	494 (27.7)	1431	432 (30.1)	N/A	N/A
Unemployment	1779	198 (11.0)	1430	101 (7.1)	1133	43 (3.8)
Retirement	1779	1142 (64.1)	1430	1011 (70.7)	1133	852 (75.1)
Hypertension^a^	1779	1324 (74.4)	1426	1084 (76.02)	1041	736 (70.7)
Diabetes (self-reported)	1778	274 (15.4)	1431	258 (18.03)	1130	216 (19.1)
Chronic heart failure	1688	163 (9.6)	1222	90 (7.3)	1112	158 (14.2)
Antihypertensive medication	1779	985 (55.3)	1426	920 (64.5)	1132	830 (73.3)
Lipid lowering medication	1779	295 (16.5)	1426	347 (24.3)	1132	308 (27.2)
Anti-diabetic medication	1779	218 (12.2)	1426	203 (14.2)	1132	181 (15.9)

*N/A* not available, *BMI* body mass index, *SBP* systolic blood pressure, *DBP* diastolic blood pressure, *HDL* high density lipoprotein, *LDL* low density lipoprotein, *HbA1c* hemoglobin A1c, *eGFR* estimated glomerular filtration rate, *CRP* C-reactive protein

^a^Measured blood pressure > 140/90 or intake of antihypertensive medication

### Follow‑up examinations

After a mean of 4.0 years (standard deviation [SD] = 0.3), 1436 (86%) subjects took part in the first follow-up examination. A second follow-up was performed after a mean of 8.8 years (SD = 0.7) with 1136 (77%) participants. Information on the vital status of participants is updated before the beginning of each follow-up and irregularly in between the follow-ups via a query at the residents' registration office. For deceased participants, a copy of the official death certificate is requested from the local health authority. The cause of death was defined as specified in the official death certificate compiled by the Federal Statistical Office. Initially, the cause of death was recorded by a medical doctor and subsequently reviewed by a certified coder at the Statistical State Office Saxony-Anhalt.

The third follow-up examination within the BioSALSA project (Biomarkers for Healthy Aging in Saxony-Anhalt) is currently taking place and will be finished by the end of 2021. For participants who are unable to come to the study center due to illness or fragility and whose address of residence was known, home visits with a reduced examination protocol are performed as in the first two follow-ups.

### Data collection

Investigations performed at baseline, first follow-up and the current third follow-up included a standardized computer-assisted personal face-to-face interview, self-administered questionnaires, an extensive medical examination, and drawing of non-fasting venous blood samples (Tables 2, 3, 4). In the second follow-up, only an interview, self-administered questionnaires, blood pressure measurements, and anthropometry were performed. Standard operating procedures have been defined for each clinical examination which were then used at every follow-up. Interview and medical examinations were performed by trained and certified study nurses. At baseline and third-follow-up, echocardiographic examinations were conducted and evaluated by a specially trained and certified physician. At the first follow-up, echocardiography was performed by a trained and certified study nurse, and subsequently the stored echocardiographic recordings were evaluated by a trained physician. All echocardiographers underwent the same dedicated study certification procedures. The self-administered questionnaires were given to the participants to be filled at home. The baseline and first follow-up interview and examination took place at the previous CARLA study center in the University Hospital of the Martin-Luther-University Halle-Wittenberg. The second and ongoing third follow-up examinations are conducted at the current CARLA study center of the Institute of Medical Epidemiology of the Martin-Luther-University Halle-Wittenberg. The average duration was 3.5 h per participant.

**Table 2:** Interview and questionnaire conducted at baseline (CARLA-0), first (CARLA-1), second (CARLA-2) and third (CARLA-3) follow-up examination

Instrument	Time-points			
	CARLA-0	CARLA-1	CARLA-2	CARLA-3
*Interview*				
Sociodemographic factors [16]	x	x	x	x
Utilization of medical services [14]	x	x	x	x
Medical history (self-reported) [13–15]	x	x	x	x
Family history [13–15]	x	x	–	–
Menopausal state and use of hormone replacement therapy	x	x	x	–
Medication use during the preceding 7 days [19]	x	x	x	x
Smoking, alcohol, and physical activity [14, 16, 17]	x	x	x^a^	x^a^
Food questionnaire [20]	x	x	x^a^	x^a^
Material circumstances [27]	x	x	x	x^a^
Social support [23]	x	x	x	x
Employment and personal financial situation after German reunification in 1990	x	–	–	–
Unemployment, job insecurity [14, 27]	x	x	–	–
Health literacy [26]	–	–	x	–
*Questionnaires*				
Health-related quality of life (SF-12) [21, 22]	x	x	x	x
Perceived morale/attitude toward own aging [69]		x	x	x
Hostility [70]	–	x	–	–
Social networks [23]	x	x	x	x
Job strain, effort-reward imbalance [71]	x	x	–	–
Depression scale [25]	x	x	x	x
Perceived security in neighborhood environments before and after German reunification in 1990	x	–	–	–
Activities of daily Life (ADL) [24]		x	x	x
Anxiety [46]	–	–	–	x
Resilience [44]	–	–	–	x
Cognitive Reserve [45]	–	–	–	x
Alcohol use—binge drinking [27]	x	–	–	–
Health beliefs	x	–	–	–
Sleep disorder	–	x	–	–

^a^As part of the questionnaires

**Table 3:** Physical and medical examinations conducted and collected biosamples at baseline (CARLA-0), first (CARLA-1), second (CARLA-2) and third (CARLA-3) follow-up examination

Instrument	Time-points			
	CARLA-0 (N = 1779)	CARLA-1 (N = 1436)	CARLA-2 (N = 1140)	CARLA-3 (N = ongoing)
*Anthropometry*				
Body weight and body height (standing) [15, 72]	x	x	x	x
Waist and hip circumference	x	x	x	x
Body impedance analysis (BIA) [72]	–	–	–	x
3D Body Scanner [72]	–	–	–	x
*Cardiovascular system*				
Blood pressure [15, 28]	x	x	x	x
Echocardiography, systolic and diastolic function	x	x	–	x
12-leads Electrocardiogram (20-min and 10-s) [30, 31]	x	x	–	x^a^
Ankle–brachial index (ABI) [36)]	x	x	–	x
Pulse wave analysis (PWA)		x^b^		x^b^
AGE reader (skin autofluorescence)	–	x (N = 368)	–	x
*Cognitive function*				
Mini Mental State Examination	–	x^c^ (N = 1020)	–	x
Semantic memory, episodic memory, working memory [35, 36]	–	–	–	x
Numerical reasoning (fluid intelligence), passive vocabulary (crystallized intelligence) [35]	–	–	–	x
Attention/executive, motor coordination [35]	–	–	–	x
*Gait and balance assessment*				
Timed up and Go Test (TMUG) [39]	–	–	–	x
Gait analysis using portable analysis system (Rehagait system) [40]	–	–	–	x
Balance assessment using pressure distribution measurement system (Zebris FM System) [41]	–	–	–	x
*Sensory organ*				
Olfactory test (Sniffin’ sticks 12) [38]	–	–	–	x
*Bio-specimens*				
Serum	x	x	–	x
EDTA whole blood	x	x	–	–
EDTA plasma	x	x	–	x
EDTA cellular components	x	x	–	x
MPA-stabilized EDTA plasma	x	x	–	–
Heparin plasma	–	x	–	x
Citrate plasma	x	x	–	x
Peripheral blood mononuclear cells	–	–	–	x
Urine	–	x	–	x
Stabilized stool	–	–	–	x
*Physical activity, physical fitness*				
Hand grip strength [42]	–	–	–	x
7-Day accelerometry [43]	–	–	–	x

^a^20-min ECG was not performed. Instead, a 5-min ECG was performed

^b^SphygmoCor device (CARLA-1) Vascular Explorer (CARLA-3)

^c^Only in participants >  = 60yrs

**Table 4:** Blood and urine parameters determined at baseline (CARLA-0), first (CARLA-1) and third (CARLA-3) follow-up examination

Parameter	CARLA-0	CARLA-1	CARLA-3
*Plasma and serum*			
Erythrocytes (RBC)	x	x	x
Nucleated red blood cells	–	–	x
Hemoglobin	x	x	x
Hematocrit	–	x	x
Leucocytes (WBC)	x	x	x
Lymphocytes	–	x	x
Neutrophilic granulocytes	–	x	x
Eosinophilic granulocytes	–	x	x
Basophilic granulocytes	–	x	x
Immature granulocytes	–	–	x
Monocytes	–	x	x
Platelets	–	x	x
Mean corpuscular volume	–	x	x
Mean corpuscular haemoglobin	–	x	x
Mean corpuscular haemoglobin concentration	–	x	x
Red cell distribution width	–	x	x
Mean platelet volume	–	x	–
Glucose	x	x	x
Glycated hemoglobin (Hba1c)	x	x	x
Creatinine	x	x	x
Triglycerides	x	x	x
Total cholesterol	x	x	x
High density lipoprotein cholesterol	x	x	x
Low density lipoprotein cholesterol (LDL)	x	x	x
Small dense LDL	–	x	–
Apolipoprotein A1	–	x	x
Apolipoprotein B	–	x	x
Alanine aminotransferase	x	x	x
Aspartate aminotransferase	x	x	x
Gamma glutamyl transferase	x	x	x
α-Amylase	x	–	x
Lipase	x	–	x
N-terminal pro-brain-natriuretic peptide	x	x	x
(High-sensitivity) C reactive protein	x	x	x
Interleukin 1β (IL-1)	x	–	–
Interleukin 6 (IL-6)	x	x	x
Interferon γ	x	–	–
Tumor necrosis factor α (TNF-α)	x	–	–
soluble TNF-receptor 1 (sTNF-R1)	x	x	–
soluble TNF-receptor 2 (sTNF-R2)	x	–	–
Albumin	–	–	x
Insulin-like growth factor-1	x^a^	–	x
Dehydroepiandrosterone sulfate	x^a^	–	x
Testosterone	x^a^	–	x
Prostate specific antigen (m)	x^a^	–	x
Ferritin (f)	x^a^	–	x
Sex hormone binding globulin	x^a^	–	x
Cystatin C	x^a^	–	x
Thyroid stimulating hormone	x	–	–
Free triiodothyonine	x	–	–
Total triiodothyonine	x	–	–
Free thyroxine	x	–	–
Total thyroxine	x	–	–
Antithyroglobulin antibodies	x	–	–
Antithyroperoxidase antibodies	x	–	–
Folate	x	–	–
Vitamin B12	x	–	–
Soluble RAGE	x	x^a^	x^a^
AGE-specific fluorescence	x	x^a^	x^a^
Influenza A IgG			x^a^
Influenza B IgG			x^a^
CMV IgG/IgM			x^a^
Serum metabolites measured by targeted metabolomics AbsoluteIDQ p150 Kit	x	x	
*Urine*			
Dip stick (bilirubin, erythrocytes, glucose, ketone, leucocytes, nitrite, pH, protein, spec. weight, urobilinogen)	x	x	x
Sediment (bacteria, erythrocytes, leucocytes)	–	–	x
Creatinine	–	–	x
Albumin	–	–	x
Uric acid	–	–	x

^a^Parameters determined from frozen samples as part of the third follow-up

### Interview and questionnaires

The standardized, computer-assisted interview collected information regarding medical history (based on [13–15]), sociodemographic and socioeconomic variables (based on [16]), utilization of medical services (based on [14]), psychosocial and lifestyle factors (based on [14, 15, 17, 18]) (Table 2). The use of medication within the 7 days preceding examination was documented using the IDOM software to derive ATC codes [19]. Additionally, self-administered questionnaires were employed which included validated tools: a food frequency questionnaire adapted from / as in the EPIC Potsdam Study Follow-up [20], questionnaires on quality of life (based on [21, 22]), the adapted German version of the Berkman scale of social support and social networks [23], information on instrumental activities of daily living was collected based on a modified questionnaire from Lawton and Brody [24], physical activity was documented using the adapted Baecke questionnaire [17], smoking was recorded according to the recommendations of the German Epidemiological Association [16], depressed mood using the Center for Epidemiologic Studies Depression Scale (CES-D) (based on [25]), and health literacy as described by Sorensen [26]. Questionnaires on alcohol consumption, unemployment and job insecurity and material circumstances were adapted from the Study of Health In Pomerania (SHIP) [14] and the Health, Alcohol and Psychosocial factors in Eastern Europe (HAPIEE) [27] Study, respectively. Some elements were implemented in the interview at baseline and first follow-up and then were applied as self-administered questionnaires in the second and third follow-up (Table 2).

### Examinations

The following examinations and measurements (Table 3) have been carried out in the CARLA Cohort:

#### Anthropometric parameters

The anthropometric measurements followed the procedures used in the MONICA/KORA and SHIP study [14, 15]. Weight and height were measured with the SECA 701 (seca GmbH & co.kg, Halle, Germany) digital scale and the SECA 220 (seca GmbH&Co.KG, Halle, Germany) height measuring system. Waist and hip circumference were measured using a flexible tape, with the study subject standing in front of a full-sized mirror, which allowed checking the horizontal position of the tape. Weight was recorded with a precision of 100 g, and height, waist and hip circumference to the nearest 0.1 cm.

#### Blood pressure measurement

After a resting period of at least five minutes, the measurement of systolic and diastolic blood pressure was performed. Blood pressure was measured with the OMRON HEM-705CP automated oscillometric blood pressure device [28] according to the procedure employed in the SHIP and KORA/MONICA Study [14, 29]. Three measurements were conducted on the left arm with a three-minute interval between measurements. The heart rate was counted manually during the resting time. In the third follow-up, we use the OMRON HEM-705IT, which is the successor device of OMRON HEM-705CP.

#### Electrocardiogram (ECG) recording

Two resting ECGs were recorded: one 10 s and one 20 min (5 min in third follow-up) 12-lead ECG. The participants were in a supine resting position for at least 20 min before the recording of the ECG began. Throughout the 20-min respectively the 5-min ECG, participants were instructed to breathe at a rate of 15/min (0.25 Hz). This was done to standardize the ECG recording with respect to the influence of the respiratory rate on the determination of spectral parameters of the heart rate variability (HRV). All 10-s ECGs were processed by the Modular ECG Analysis System (MEANS) [30] to obtain Minnesota Codes [31]. MEANS was additionally used to process the 20-min/5-min ECGs to obtain the locations and types of the QRS complexes, which were then used to compute standard time domain and frequency domain parameters of HRV. The method of computing HRV has been described in detail elsewhere [8, 32, 33].

#### Echocardiographic measurements

The analysis included parameters of left ventricular dimension (left ventricular mass) and of systolic and diastolic function derived from M-Mode and Doppler echocardiographic measurements.

Echocardiographic examinations at baseline and first follow-up were performed using the GE Vivid ultrasound system (GE Vivid 4 and 5 at baseline, GE vivid 5 at first follow-up). At the third follow-up, a Philips iE33 3D-echocardiographic device is used. Echocardiographic images are obtained in standardized parasternal and apical views using 2D, M-Mode, pulse wave and tissue doppler imaging. Echocardiographic measurements included parameters of left ventricular dimension, systolic and diastolic function. All acquired images and media are stored on a secured network server as digital clips, using a unique identification number, and analyzed on a dedicated workstation (EchoPAC PC, version 110.1.0, GE Healthcare and Philips IntelliSpace Cardiovascular 3.2).

#### Ankle–brachial index (ABI)

For ABI measurement in CARLA at baseline and first follow-up, the supine systolic blood pressure (BP) at the arm and ankle was measured after 5 min of rest using the OMRON HEM-705CP device. Measurement of BP was started simultaneously on the arm and ankle. Two measurements were conducted on the right ankle, followed by two measurements on the left ankle with a one-minute delay between each pair of measurements. To calculate the ABI, the mean systolic BP of that ankle side which was lowest was divided by the brachial systolic BP which was the highest. Further details are described elsewhere [34]. In the third follow-up, the Vascular Explorer (VaE) (Fa. Enverdis GmbH, Jena, Deutschland) is used to measure ABI as adapted from the NAKO study (National Cohort Study) [35]. For the determination of the ABI, the blood pressure is measured with the help of oscillometric blood pressure cuffs at both arms and legs as well as so-called occlusion pressure values at all four extremities through photo-plethysmographic measurement methods (SPO2 sensors).

#### Pulse wave analysis (PWA)

PWA was performed noninvasively in the first follow-up by applanation tonometry of the radial artery using SphygmoCor. During the third follow-up, PWA was perfomed using the VaE, according to the procedure employed in the NAKO Study [35, 36]. The following parameters of the vascular stiffness are determined via algorithms: pulse wave velocity, augmentation index and aortic blood pressure.

#### Advanced glycation end products (AGEs) analysis

Skin autofluorescence induced by the formation of AGEs was recorded in the first follow-up in a subgroup of N = 368 and will be measured in all participants in the third follow-up with the AGE Reader SU (DiagnOptics, The Netherlands).

### Additional examinations in the third follow-up

In the third follow-up, our aim is to focus on aging processes from the perspective of a healthy aging (within the BioSALSA project: Biomarkers for Healthy Aging in Saxony Anhalt). It is envisaged that parameters of physiological, neurocognitive and immunological functioning as well blood biomarker profiles will allow a characterization of the (healthy) ageing process of the participants and their cardiovascular system. In addition to the examinations that were conducted at baseline and first follow-up, several further examinations are undertaken, such as the cognitive function test battery adapted from the German National Cohort (NAKO) [35, 36], an olfactory test [38], gait assessment using Timed up and Go test [39], an inertial-sensor based device [40], balance assessment using a pressure distribution plate [41], as well as the assessment of physical fitness using hand grip strength test [37, 42] and a 7-day accelerometry [43]. Using self-administered questionnaires, we will also measure psychosocial factors affecting ageing, such as resilience [44], cognitive reserve [45], depressed mood [25], and anxiety [46].

### Laboratory analyses

#### Biosamples

Non-fasting, venous blood samples as well as other biomaterials were collected and processed following the standardized operating procedures of each examination by trained laboratory personnel at baseline and at the first follow-up. In general, samples were centrifuged, separated and immediately placed on ice for processing. Aliquoted samples were stored at -80 °C for future analyses. Detailed information about the collected biospecimens in each examination of the participants is given in Table 4.

In the third follow-up, Ethylenediaminetetraacetic acid (EDTA), citrate and heparin stabilized plasma as well as EDTA cellular components, serum, urine and stabilized stool will be collected and stored at − 80 °C to allow broad subsequent analyses. Furthermore, peripheral blood mononuclear cells (PBMCs) will be isolated by FICOLL gradient centrifugation utilizing BD Vacutainer CPT sodium citrate stabilized plasma tubes. Therefore, CPT vacutainers are centrifuged for 22 min at 1700×*g*, sodium citrate plasma is collected, replaced by phosphate-buffered saline (PBS) and the PBMCs are then decanted into 15 mL screw-cap tubes. After two washing steps with PBS, PBMCs are dissolved in freezing medium, aliquoted and stored at − 150 °C. The collected, corresponding sodium citrate plasma is also stored, allowing for direct pair-wise analyses of fluid and cells.

#### Analyses

An overview on the determined blood and urine parameters in baseline and first follow-up, as well as on the planned parameters of the third follow-up, is given in Table 4.

In the baseline examination, as well as in the first follow-up, blood samples were directly analyzed for a wide range of parameters including a hemogram, glucose, HbA1c, cholesterol levels, liver and pancreas enzymes, inflammatory markers, as well as kidney and thyroid function, as described before [7]. Additionally, advanced glycation end products (AGE)-specific fluorescence and their soluble receptor (sRAGE) levels were determined for non-fasting plasma samples collected at baseline [47]. Soluble tumor necrosis factor receptor R1 (TNF-R1) was determined in duplicates in serum samples of the baseline and the first follow-up using an antibody-based assay (Quantikine ELISA, R&D Systems) on an Epoch 2 Microplate Spectrophotometer [48].

Metabolite quantification was performed in the Genome Analysis Center at the Helmholtz Zentrum München using the Absolute*IDQ*™ p150 Kit (Biocrates Life Sciences AG, Austria). In more detail, a panel of 163 metabolites that includes free carnitine, 40 acylcarnitines, 14 amino acids, hexoses (sum of hexoses), 92 glycerophospholipids, and 15 sphingolipids was quantified by flow injection analysis-tandem mass spectrometry (FIA-MS/MS). The sample preparation and mass spectrometric measurements, as well as the metabolite nomenclature, have been described previously [49, 50].

In the third follow-up, measurements of most previously assessed blood parameters, as well as of additional blood and urine-parameters, are planned (Table 4). All measurements will be carried out in cooperation with the Central Laboratory of the University Hospital Halle (Saale) on Roche Cobas, Sysmex or ABL90 Flex Plus analytical platforms except for soluble RAGE and Influenza and CMV-reactive antibodies, which are determined manually by ELISA.

## Findings to date

Results from the CARLA Study have been reported in 82 publications, out of which 34 publications were using only the CARLA cohort data and 48 publications were based on collaborations between CARLA and other national and international cohorts. More details are provided in the supplementary material. The in-depth data collection has been used for describing the prevalence and incidence of cardiovascular risk factors and diseases as well as for exploratory analyses on associations between cardiovascular risk factors and various phenotypes during a period spanning 11 years. Overall, we found a very high prevalence of all classic life-style-related cardiovascular risk factors and diseases, e.g. hypertension, overweight, diabetes mellitus, and heart failure. These findings were extraordinary even with respect to other population-based epidemiologic studies in Germany.

Key publications have reported the following specific findings:

- Heart rate variability was shown to have a weak and inconsistent association with cardiovascular risk factors [8, 51, 52].
- There was a high prevalence of symptomatic chronic heart failure. Women were more affected by heart failure with preserved ejection fraction, while men suffered from heart failure with reduced ejection fraction [12].
- A decrease in systolic and diastolic blood pressure was observed between baseline and subsequent follow-ups, accompanied by an increase in anti-hypertensive medication consumption and a higher awareness of the condition. This decrease has been ascribed to a better hypertension control due to raised awareness of participants’ hypertension status after participation in the Study [53].
- We showed that a higher health literacy score was associated with different health-related outcomes even after adjustment for educational level [54].
- We found a direct association of the inflammation biomarker sTNF-R1 in the elderly male general population with renal failure development [55] and with echocardiographic parameters for ventricular hypertrophy [56], in addition to cardiovascular and all-cause mortality [48].
- Recently, we showed that AGEs and their soluble receptor sRAGE measured in plasma samples were associated with limitations in physical functioning in women, but not in men [57]. However, we couldn’t find any association of plasma AGEs and sRAGE with overall or cardiovascular mortality [58].
- We showed sex- specific effects of lifestyle risk factors [50] and ageing [59] on human metabolites. We also showed that the instability in an individuals’ own ‘metabolic space’ (metabotype) was associated with incident cardiovascular disease and all-cause mortality [60] thus, identifying metabotype instability represents a valuable indicator of pre-clinical disease.

Additionally, the data from the CARLA Study have been used in several pooled cohort analyses, such as the DIAB-CORE Consortium, which published findings on regional differences for diabetes mellitus in Germany [61] and describing the need for better blood pressure [62] and lipid-level management for subjects with diabetes [63]. The CARLA study was compared to other population-based cohorts in Germany to examine regional differences and disparities. For example, an analysis showed that the odds of type 2 diabetes prevalence were highest in the east (odds ratio = 1.98, 95% confidence interval: 1.81, 2.14) and northeast of Germany and decreased to the southwest after adjustment for individual variables [64]. In another analysis examining the incidence of type 2 diabetes, the results of an analysis of data from five population-based cohorts in Germany show that the regional incidence was highest in the East and lowest in the South of Germany with 16.9 (95% CI 13.3–21.8) vs 9.3 (95% CI 7.4–11.1)/1000 person-years, respectively [65]. The waist circumference as a measure of central obesity was also shown to be on average 3.4 and 6.7 cm higher among men and women with similar BMI in CARLA compared to a cohort from Bavaria [66].

In several genome-wide association studies, CARLA acted as a replication cohort, identifying 18 loci associated with CRP levels [67] and 4 loci associated with thyroid function [68]. The epidemiological lesson learned from a small cohort study, such as the CARLA cohort, is—when the primary goal is to examine real-life exposures, subject-related outcomes and their change, the focus should be on measures that can be standardized and replicated.

## Strengths and limitations

The main strength of the CARLA Study is the comprehensive set of important clinical, biochemical, and lifestyle information collected during a long follow-up period in a representative sample of the elderly general population. The CARLA study is one of the last epidemiological studies in Germany that aimed for representativeness to the general population and indeed achieved response proportions of more than 60%. Nevertheless, we had to discuss problems and effects of selection bias on at least partly unexpected results. An analysis of non-responders (not shown here) indicated that selection bias due to non-participation led to an underestimation of disease prevalence and of risk factors in study participants as compared to the general population, which could have biased the associations of risk factors and disease towards the null as a result of a loss of highly susceptible subjects. The data has been used for answering several research questions in cardiology, endocrinology, and genetics. The stored bio-samples allow additional analyses. The harmonization of data collection with other cohort studies (KORA, SHIP, RECALL, EPIC Potsdam and HAPIEE) allows for pooled data analyses by including individual data of multiple cohorts. Self-reported information on a physician diagnosis of heart attack, stroke, cancer and diabetes were validated by general practitioners. The current data collection expands the focus into physical functioning in old age and mental health.

The main limitation is the relatively small sample size, making it difficult to study incident disease outcomes. Moreover, blood samples were not collected during the second follow-up period, and for examinations that are performed for the first time in CARLA subjects during the current third follow-up (e.g. gait and balance test), no preceding values exist.

## Supplementary Information

Below is the link to the electronic supplementary material.

Supplementary file 1 (DOCX 32 KB)

## Data Availability

Researchers interested in a potential collaboration can apply for the data by sending an email to [carlastudie@uk-halle.de] or through submitting a form that is available on the CARLA study website https://webszh.uk-halle.de/carla-studie/. To access the data, a formal application must be submitted with a detailed research proposal consisting of a title, authors, research questions, brief scientific background, list of needed variables, and proposed statistical analyses. All proposals will be reviewed by the CARLA Study steering committee and a final decision on the use of data will be given.
