# Supplementary material for: Cardiovascular risk factors, living and ageing in Halle: the CARLA study
Source: Eur J Epidemiol. 2022 Jan 3;37(1):103–16. doi: 10.1007/s10654-021-00824-7 (PMC8791893; doi:10.1007/s10654-021-00824-7)
Supplement: Supplementary file 1 — (DOCX 32 KB) [file 10654_2021_824_MOESM1_ESM.docx]

**Cohort Profile: CA**rdiovascular **R**isk Factors, **L**iving and **A**geing in Halle **(CARLA)**

Lamiaa Hassan^1,7^*, Ljupcho Efremov^1,3,7^*, Anne Großkopf^2*^, Nadja Kartschmit^1,7^, Daniel Medenwald^3^, Artjom Schott^4^, Andrea Schmidt-Pokrzywniak^1,7^, Maria E. Lacruz^1^, Daniel Tiller^1, 8^, F. Bernhard Kraus^5^, Karin H. Greiser^6^, Johannes Haerting^1^, Karl Werdan^4^, Daniel Sedding^4^, Andreas Simm^2^, Sebastian Nuding^4^, Alexander Kluttig^1,7^#+, Rafael Mikolajczyk^1,7^#

^1^Institute of Medical Epidemiology, Biostatistics, and Informatics, Medical Faculty of the Martin-Luther-University Halle-Wittenberg, Halle (Saale), Germany

^2^University Clinic and Outpatient Clinic for Cardiac Surgery, Middle German Heart Centre at the University Hospital Halle, Halle, Germany

^3^Department of Radiation Oncology, University Hospital Halle (Saale), Halle (Saale), Germany

^4^Department of Internal Medicine III, University Hospital, Martin-Luther-University Halle-Wittenberg, Halle (Saale), Germany

^5^Central Laboratory, University Hospital Halle, Halle (Saale), Germany

^6^German Cancer Research Center, Division of Cancer Epidemiology, Heidelberg, Germany

^7^ Interdisciplinary Center for Health Sciences, Medical Faculty of the Martin-Luther-University Halle-Wittenberg, Halle (Saale), Germany

^8^ Clinical Computing Center – Data Integration Center, University Hospital Martin-Luther-University Halle-Wittenberg, Halle (Saale), Germany

*joint first-authors,

# joint last authors

+ corresponding author: alexander.kluttig@uk-halle.de

- Data supplement -

Published studies using only the CARLA cohort data

1. Greiser KH, Kluttig A, Schumann B, Kors JA, Swenne CA, Kuss O, Werdan K, Haerting J: Cardiovascular disease, risk factors and heart rate variability in the elderly general population: Design and objectives of the CARdiovascular disease, Living and Ageing in Halle (CARLA) Study. BMC Cardiovascular Disorders 2005, 5:33
2. Kors JA, Swenne CA, Greiser KH: Cardiovascular disease, risk factors, and heart rate variability in the general population. J Electrocardiol 2007; 40 (1 Suppl):S19-S21.
3. Kuss O, Schumann B, Kluttig A, Greiser KH, Haerting J: Time domain parameters can be estimated more precisely than frequency domain parameters in the analysis of heart rate variability. J Electrocardiol 2008;41:287-91.
4. Greiser KH, Kluttig A, Schumann B, Swenne CA, Kors JA, Kuss O, Haerting J, Schmidt H, Thiery J, Werdan K. Cardiovascular diseases, risk factors and short-term heart rate variability in an elderly general population: the CARLA study 2002-2006. Eur J Epidemiol. 2009; 24:123-42.
5. Kluttig A, Kuss O, Greiser KH. Ignoring lack of association of heart rate variability with cardiovascular disease and risk factors. International Journal of Cardiology 2010 Nov 19;145(2):375-6.
6. Schumann B, Seidler A, Kluttig A, Werdan K, Haerting J, Greiser KH. Association of occupation with prevalent hypertension in an elderly East German population: an exploratory cross-sectional analysis. nt Arch Occup Environ Health. 2011 Apr;84(4):361-9.
7. Kluttig A, Schumann B, Swenne CA, Kors JA, Kuß O, Schmidt H, Werdan K, Haerting J, Greiser KH. Association of Health Behaviour with Heart Rate Variability: A Population-Based Study BMC Cardiovascular Disorders 2010 Nov 25;10:58.
8. Schumann B, Kluttig A, Tiller D, Werdan K, Haerting J, Greiser KH. Association of childhood and adult socioeconomic indicators with cardiovascular risk factors and its modification by age - The CARLA Study 2002-2006. BMC Public Health 2011, 11:289.
9. Bartling B, Vanhooren V, Chen CC, Libert C, De-Waeleb S, Hofmann HS,Haerting J, Nuding S, Silber RE, Simm A. N-glycan profile of plasma proteins is altered in patients with non-small cell lung carcinoma. Cancer Biomark. 2011-2012;10(3-4):145-54
10. Haerting J, Kluttig A, Greiser KH, Nuding S, Werdan K. Kohortenstudie zu Risikofaktoren für Herz-Kreislauf-Krankheiten in einer urbanen älteren ostdeutschen Allgemeinbevölkerung (CARLA-Studie). Bundesgesundheitsbl 2012. 55:795–800.
11. Hartwig S, Kuss O, Tiller D, Greiser KH, Schulze MB, Dierkes J, Werdan K, Haerting J, Kluttig A. Validation of the German Diabetes Risk Score within a population-based representative cohort. Diabet Med. 2013 Sep;30(9):1047-53.
12. Tiller D, Russ M, Greiser KH, Nuding S, Ebelt H, Kluttig A, Kors JA, Thiery J, Bruegel M, Haerting J, Werdan K. Prevalence of symptomatic heart failure with reduced and with normal ejection fraction in an elderly general population - the CARLA Study. PLoS One. 2013;8(3):e59225.
13. Medenwald D, Dietz S, Tiller D, Kluttig A, Greiser KH, Loppnow H, Thiery J, Nuding S, Russ M, Fahrig A, Haerting J, Werdan K. Inflammation and echocardiographic parameters of ventricular hypertrophy in a cohort with preserved cardiac function. Open Heart 2014; Feb 8;1(1).
14. Medenwald D, Kors JA, Loppnow H, Thiery J, Kluttig A, Nuding S, Tiller D, Greiser KH, Werdan K, Haerting J. Inflammation and prolonged QT time: Results from the CARdiovascular disease, Living and Ageing in Halle (CARLA) study. PLoS One. 2014 Apr 25;9(4):e95994.
15. Medenwald D, Girndt M, Loppnow H, Kluttig A, Nuding S, Tiller D, Thiery JJ, Greiser KH, Haerting J, Werdan K. Inflammation and Renal Function after a Four-Year Follow-Up in Subjects with Unimpaired Glomerular Filtration Rate: Results from the Observational, Population-Based CARLA Cohort. PLoS One. 2014 Sep 26;9(9).
16. Kussmaul T, Greiser KH, Haerting J, Werdan K, Thiery J, Kratzsch J. Thyroid analytes TSH, FT3 and FT4 in serum of healthy elderly subjects as measured by the Roche modular system: do we need age and gender dependent reference levels? Clin Lab. 2014;60(9):1551-9.
17. Medenwald D, Kluttig A, Kors JA, Nuding S, Tiller D, Greiser KH, Werdan K, Haerting J. QT interval, general mortality and the role of echocardiographic parameters of left ventricular hypertrophy: Results from the prospective, population-based CARLA study. Eur J Prev Cardiol. 2015 May 21.
18. Lacruz E, Kluttig A, Hartwig S, Löer M, Tiller D, Greiser KH, Werdan K, Haerting J. Prevalence and incidence of hypertension in the general adult population - Results of the CARLA-cohort study. Medicine 2015 Jun;94(22):e952.
19. Schröder J, Nuding S, Müller-Werdan U, Werdan K, Kluttig A, Russ M, Greiser K, Kors J, Haerting J, Medenwald D. Performance of Sokolow-Lyon index in detection of echocardiographically diagnosed left ventricular hypertrophy in a normal Eastern German population - results of the CARLA study. BMC Cardiovascular Disorders 2015. 15:69.
20. Medenwald D, Loppnow H, Kluttig A, Nuding S, Greiser KH, Thiery J, Tiller D, Herzog B, Werdan K, Haerting J. Educational level and chronic inflammation in the elderly - the role of obesity: results from the population-based CARLA study. Clin Obes. 2015 Jul 31.
21. Tiller D, Herzog B, Kluttig A, Haerting J. Health literacy in an urban elderly East-German population - results from the population-based CARLA study. BMC Public Health. 2015 Sep 10;15(1):883.
22. Lacruz E, Tiller D, Kluttig A, Greiser KH, Nuding S, Werdan K, Haerting J Association of late-life changes in blood pressure and cognitive status. J Geriatr Cardiol. 2016 Jan; 13(1): 37–43.
23. Bohley S, Kluttig A, Werdan K, Nuding S, Greiser KH, Kuß O, Markus M, Schmidt CO, Völzke H, Krabbe C, Haerting J. Changes of individual perception in psychosocial stressors related to German reunification in 1989/1990 and cardiovascular risk factors and cardiovascular diseases in a population-based study in East Germany. BMJ Open. 2016; 6(1): e008703.
24. Lacruz ME, Kluttig A, Kuss O, Tiller D, Medenwald D, Nuding S, Greiser KH, Frantz S, Haerting J. [Short-term blood pressure variability - variation between arm side, body position and successive measurements: a population-based cohort study.](https://www.ncbi.nlm.nih.gov/pubmed/28100183)BMC Cardiovasc Disord. 2017 Jan 18;17(1):31
25. Lacruz ME, Kluttig A, Tiller D, Medenwald D, Giegling I, Rujescu D, Prehn C, Adamski J, Frantz S, Greiser KH, Emeny RT, Kastenmüller G, Haerting J. [Cardiovascular Risk Factors Associated With Blood Metabolite Concentrations and Their Alterations During a 4-Year Period in a Population-Based Cohort.](https://www.ncbi.nlm.nih.gov/pubmed/27784734) Circ Cardiovasc Genet. 2016 Dec;9(6):487-494.
26. Medenwald D, Tiller D, Nuding S, Greiser KH, Kluttig A, Frantz S, Haerting J. [Educational status and differences in left ventricular mass and ejection fraction - The role of BMI and parameters related to the metabolic syndrome: A longitudinal analysis from the population-based CARLA cohort.](https://www.ncbi.nlm.nih.gov/pubmed/27397510) Nutr Metab Cardiovasc Dis. 2016 Sep;26(9):815-23.
27. Medenwald D, Swenne CA, Loppnow H, Kors JA, Pietzner D, Tiller D, Thiery J, Nuding S, Greiser KH, Haerting J, Werdan K, Kluttig A. [Prognostic relevance of the interaction between short-term, metronome-paced heart rate variability, and inflammation: results from the population-based CARLA cohort study.](https://www.ncbi.nlm.nih.gov/pubmed/27221352) Europace. 2017 Jan;19(1):110-118.
28. Lacruz ME, Kluttig A, Tiller D, Medenwald D, Giegling I, Rujescu D, Prehn C, Adamski J, Greiser KH, Kastenmüller G. Instability of personal human metabotype is linked to all-cause mortality. Sci Rep. 2018 Jun 28;8(1):9810.
29. Sedlmeier A, Kluttig A, Giegling I, Prehn C, Adamski J, Kastenmüller G, Lacruz ME. The human metabolic profile reflects macro- and micronutrient intake distinctly according to fasting time. Sci Rep. 2018 Aug 16;8(1):12262.
30. Medenwald D, Kluttig A, Lacruz ME, Schumann J. Serum dietary fatty acids and coronary heart disease risk - A nested case-control-study within the CARLA cohort. Nutr Metab Cardiovasc Dis. 2019 Feb;29(2):152-158.
31. Ebert H, Lacruz ME, Kluttig A, Simm A, Greiser KH, Tiller D, Kartschmit N, Mikolajczyk R. Advanced glycation end products and their ratio to soluble receptor are associated with limitations in physical functioning only in women: results from the CARLA cohort. BMC Geriatr. 2019 Nov 4;19(1):299. doi: 10.1186/s12877-019-1323-8.
32. Ebert H, Lacruz ME, Kluttig A, Simm A, Greiser KH, Tiller D, Kartschmit N, Mikolajczyk R. Association between advanced glycation end products, their soluble receptor, and mortality in the general population: Results from the CARLA study. Exp Gerontol. 2020 Mar;131:110815. doi: 10.1016/j.exger.2019.110815. Epub 2019 Dec 16.
33. Efremov L, Lacruz EM, Tiller D, Medenwald D, Greiser KH, Kluttig A, Wienke A, Nuding S, Mikolajczyk R. Metabolically healthy, but obese individuals and associations with echocardiographic parameters and inflammatory biomarkers: results from the CARLA Study. Diabetes Metab Syndr Obes. 2020 Jul 27;13:2653-2665.
34. Hassan L, Medenwald D, Tiller D, Kluttig A; Ludwig-Kraus B, Kraus FB, Greiser KH, Mikolajczyk, R. The association between change of soluble tumor necrosis factor receptor R1 (sTNF-R1) measurements and cardiovascular and all-cause mortality–results from the population-based (Cardiovascular Disease, Living and Ageing in Halle) CARLA Study 2002–2016. PLoS One 2020 Oct 26;15(10):e0241213.

Collaborations between the CARLA Study and other national and international cohorts

1. Vasan SR et al., … Greiser KH, … Haerting J, … Werdan K,… Novel Genetic Variants Associated with Cardiac Structure and Function: Genome-wide Association Findings of a Prospective Meta-analysis from the EchoGen Consortium. JAMA 2009;302(2):168-178.
2. Tamayo T … Greiser KH, … Rathmann W für das DIAB-CORE Konsortium: Zusammenschluss von populationsbasierten Studien ermöglicht regionale Vergleiche zum Diabetes mellitus in Deutschland (DIAB-CORE Verbund). Med Welt 2010, 61(2):94-96.
3. Teupser D, …Greiser KH, … Kluttig A, Werdan K, … et al., Genetic regulation of serum phytosterol levels and risk of coronary artery disease. Circ Cardiovasc Genet. 2010 Aug;3(4):331-9..
4. Stang A, Doring A, Volzke H, Moebus S, Greiser KH, Werdan K, Berger K, Ellert U, Neuhauser H: Regional Differences in Body Fat Distributions among People with Comparable Body Mass Index. A Comparison across Six German Population-based Surveys. European Journal of Cardiovascular Prevention and Rehabilitation 2011 Feb;18(1):106-14.
5. Dehghan A, .... Werdan K, ,Greiser KH, Kuß O…, Chasman DI. Meta-analysis of genome-wide association studies in over 80,000 subjects identifies 11 novel loci for C-reactive protein levels. Circulation 2011 Feb 22;123(7):731-8.
6. Schunk M, Reitmeir P, Schipf S, Völzke H, Meisinger C, Thorand B, Kluttig A, Greiser KH, Berger K, Müller G, Ellert U, Neuhauser H, Tamayo T, Rathmann W, Holle R. Health-related Quality of Life in Subjects with and without Type 2 Diabetes: Pooled Analysis of Five Population-based Surveys in Germany. Diabet Med 2012 May;29(5):646-653.
7. Schipf S, Werner A, Tamayo T, Holle R, Schunk M, Maier W, Reitmeir P, Meisinger C, Thorand B, Berger K, Müller G, Moebus S, Bokhof B, Kluttig A, Greiser KH, Neuhauser H, Ellert U, Icks A, Rathmann W, Völzke H. Regional differences in the prevalence of known type 2 diabetes mellitus in 45-74 years old individuals: Results from six population-based studies in Germany (DIAB-CORE Consortium). Diabet Med. 2012 Jul;29(7):e88-95.
8. Rückert IM, Schunk M, Holle R, Schipf S, Völzke H, Kluttig A, Greiser KH, Berger K, Müller G, Ellert U, Neuhauser H, Rathmann W, Tamayo T, Moebus S, Andrich S, Meisinger C. Blood pressure and lipid management fall far short in persons with type 2 diabetes: Results from the DIAB-CORE Consortium including six German population-based studies. Cardiovascular Diabetology 2012 May 8;11:50.
9. Rawal R, Teumer A, Völzke H, Wallaschofski H, Ittermann T, Asvold BO, Bjøro T, Greiser KH, Tiller D, Werdan K, Meyer zu Schwabedissen HE, Doering A, Illig T, Gieger C, Meisinger C, Homuth G. Meta-analysis of two genome-wide association studies identifies four genetic loci associated with thyroid function. Hum Mol Genet. 2012 Jul 15;21(14):3275-82.
10. Maier W, Holle R, Hunger M, Peters A, Meisinger C, Greiser KH, Kluttig A, Völzke H, Schipf S, Moebus S, Bokhof B, Berger K, Mueller G, Rathmann W, Tamayo T, Mielck A (for the DIAB-CORE consortium). The impact of regional deprivation and individual socioeconomic status on the prevalence of type 2 diabetes in Germany. A pooled analysis of five population-based studies. Diabet Med. 2013 Mar;30(3):e78-86.
11. Rückert IM, Maier W, Mielck A, Schipf S, Völzke H, Kluttig A, Greiser KH, Berger K, Müller G, Ellert U, Neuhauser H, Rathmann W, Tamayo T, Moebus S, Andrich S, Meisinger C. Personal attributes that influence the adequate management of hypertension and dyslipidemia in patients with type 2 diabetes. Results from the DIAB-CORE Cooperation. Cardiovasc Diabetol. 2012 Oct 5;11(1):120.
12. Müller G, Kluttig A, Greiser KH, Moebus S, Slomiany U, Schipf S, Völzke H, Maier W, Meisinger C, Tamayo T, Rathmann W, Berger K. Regional and Neighborhood Disparities in the Risk of Type 2 Diabetes: Results from Five Population-Based Studies in Germany (DIAB-CORE Consortium). Am J Epidemiol. 2013 Jul 15;178(2):221-30.
13. Taudien S, Gäbel G, Kuss O, Groth M, Grützmann R, Huse K, Kluttig A, Wolf A, Nothnagel M, Rosenstiel P, Greiser KH, Werdan K, Krawczak M, Pilarsky C, Platzer M. Association studies of the copy-number variable ß-defensin cluster on 8p23.1 in adenocarcinoma and chronic pancreatitis. BMC Res Notes. 2012 Nov 13;5:629.
14. Ittermann T, Tiller D, Meisinger C, Agger C, Nauck M, Rettig R, Hofman A, Jørgensen T, Linneberg A, Witteman JCM, Greiser KH, Werdan K, Döring A, Kluttig A, Stricker BHC, Völzke H. High Serum TSH Levels are Associated with Current but not with Incident Hypertension. Thyroid. 2013 Aug;23(8):955-63.
15. Meisinger C, Ittermann T, Tiller D, Agger C, Nauck M, Schipf S, Wallaschofski H, Jørgensen T, Linneberg A, Thiery J, Kluttig A, Greiser KH., Werdan K, Burkhardt K, Völzke H. Gender-Specific Associations between Thyroid-Stimulating Hormone and Serum Lipid Profiles. Thyroid. 2014 Mar;24(3):424-32.
16. Medici M, …Pietzner D, Tiller D, Kluttig A, … Peeters RP. A meta-analysis of 11 genome-wide association studies identifies novel loci associated with thyroid peroxidase antibodies and clinical thyroid disease. PLoS Genet. 2014 Feb 27;10(2):e1004123.
17. Müller G, Hartwig S, Greiser KH, Moebus S, Pundt N, Schipf S, Völzke H, Maier W, Meisinger C, Tamayo T, Rathmann W, Berger K; DIAB-CORE Consortium.Gender differences in the association of individual social class and neighbourhood unemployment rate with prevalent type 2 diabetes mellitus: a cross-sectional study from the DIAB-CORE consortium. BMJ Open. 2013 Jun 21;3(6).
18. Stang A, Kluttig A, Moebus S, Völzke H, Berger K, Greiser KH, Stöckl D, Jöckel KH, Meisinger C.Educational level, prevalence of hysterectomy, and age at amenorrhoea: a cross-sectional analysis of 9536 women from six population-based cohort studies in Germany. BMC Womens Health. 2014 Jan 16;14(1):10
19. Arking DE, Pulit SL,…Kluttig A, …, Greiser KH, …, Werdan K, …, Haerting J, …, Newton-Cheh C. Genetics highlights calcium signaling pathways in myocardial repolarization. Nature Genetics 2014. Aug;46(8):826-36.
20. Schipf S, Ittermann T, Tamayo T, Holle R, Schunk M, Maier W, Meisinger C, Thorand B, Kluttig A, Greiser KH, Berger K, Müller G, Moebus S, Slomiany U, Icks A, Rathmann W, Völzke H. Regional differences in the incidence of self-reported type 2 diabetes in Germany: results from five population-based studies in Germany (DIAB-CORE Consortium). J Epidemiol Community Health. 2014 Jul 29. [Epub ahead of print]
21. Schunk M, Reitmeir P, Schipf S, Völzke H, Meisinger C, Ladwig KH, Kluttig A, Greiser KH, Berger K, Müller G, Ellert U, Neuhauser H, Tamayo T, Rathmann W, Holle R. Health-related quality of life in women and men with type 2 diabetes: a comparison across treatment groups. J Diabetes Complications. 2015 Mar;29(2):203-11.
22. Schunk M, Reitmeir P, Schipf S, Völzke H, Meisinger C, Ladwig KH, Kluttig A, Greiser KH, Berger K, Müller G, Ellert U, Neuhauser H, Tamayo T, Rathmann W, Holle R. Health-related quality of life in women and men with type 2 diabetes: a comparison across treatment groups. J Diabetes Complications. 2015 Mar;29(2):203-11.
23. Rückert IM, Baumert J, Schunk M, Holle R, Schipf S, Völzke H, Kluttig A, Greiser KH, Tamayo T, Rathmann W, Meisinger C. Blood Pressure Control Has Improved in People with and without Type 2 Diabetes but Remains Suboptimal: A Longitudinal Study Based on the German DIAB-CORE Consortium. PLoS One. 2015 Jul 29;10(7).
24. Hartwig S, Greiser KH, Medenwald D, Tiller D, Herzog B, Schipf S, Ittermann T, Völzke H, Müller G, Haerting J, Kluttig A. Association of change of anthropometric measurements with incident type 2 diabetes mellitus - a pooled analysis of the prospective population-based CARLA and SHIP studies. Medicine 2015 Aug;94(34):e1394.
25. Müller G., Wellmann J., Hartwig S., Greiser K.H., Moebus S., Jöckel K.H., Schipf S., Völzke H., Maier W., Meisinger C., Tamayo T., Rathmann W., Berger K. Association of neighbourhood unemployment rate with incident Type 2 diabetes mellitus in five German regions. DIABETIC MEDICINE 2015, 32: 1017-22.
26. Haftenberger M, Mensink GB, Herzog B, Kluttig A, Greiser KH, Merz B, Nöthlings U, Schlesinger S, Vogt S, Thorand B, Peters A, Ittermann T, Völzke H, Schipf S, Neamat-Allah J, Kühn T, Kaaks R, Boeing H, Bachlechner U, Scheidt-Nave C, Schienkiewitz A. Changes in body weight and obesity status in German adults: results of seven population-based prospective studies. Eur J Clin Nutr. 2015 Oct 28. doi: 10.1038/ejcn.2015.179.
27. Ittermann T, Lorbeer R, Tiller D, Lehmphul I, Kluttig A, Felix SB, Werdan K, Greiser KH, Köhrle J, Völzke H, Dörr M Serum Thyrotropin Concentrations Are Not Associated with the Ankle-Brachial Index: Results from Three Population-Based Studies. Eur Thyroid J. 2015 Sep;4(Suppl 1):101-7.
28. Herzog B, Lacruz ME, Haerting J, Hartwig S, Tiller D, Medenwald D, Vogt S, Thorand B, Holle R, Bachlechner U, Boeing H, Merz B, Nöthlings U, Schlesinger S, Schipf S, Ittermann T, Aumann N, Schienkiewitz A, Haftenberger M, Greiser KH., Neamat-Allah J, Katzke V, Kluttig A. Association between socioeconomic status and anthropometric changes – a meta-analytic approach from seven German cohorts. Obesity 2016 Mar;24(3):710-8.
29. Hartwig S, Kluttig A, Tiller D, Fricke J, Müller G, Schipf S, Völzke H, Schunk M, Meisinger C, Schienkiewitz A, Heidemann C, Moebus S, Pechlivanis S, Werdan K, Kuss O, Tamayo T, Haerting J, Greiser KH. Anthropometric markers and their association with incident type 2 diabetes mellitus – which marker is best for prediction? Pooled analysis of four German population-based cohort studies and comparison with a nationwide cohort study. BMJ open 2016 Jan 20;6(1)
30. Seibert E, Zohles K, Ulrich C, Kluttig A, Nuding S, Kors JA, Swenne CA, Werdan K, Fiedler R, Girndt M. [Association between autonomic nervous dysfunction and cellular inflammation in end-stage renal disease.](https://www.ncbi.nlm.nih.gov/pubmed/27809785) BMC Cardiovasc Disord. 2016 Nov 3;16(1):210.
31. Haftenberger M, Mensink GBM, Vogt S, Thorand B, Peters A, Herzog B, Hartwig S, Greiser KH, Ittermann T, Schipf S, Völzke H, Merz B, Nöthlings U, Koch M, Neamat-Allah J, Katzke V, Kaaks R, Boeing H, Bachlechner U, Scheidt-Nave C, Schienkiewitz A. Changes in Waist Circumference among German Adults over Time - Compiling Results of Seven Prospective Cohort Studies. OBESITY FACTS 2016, 9: 332-343
32. Barban N, Jansen R, de Vlaming R, … Kluttig A, … Koellinger PD, den Hoed M, Snieder H, Mills MC. [Genome-wide analysis identifies 12 loci influencing human reproductive behavior.](https://www.ncbi.nlm.nih.gov/pubmed/27798627) Nat Genet. 2016 Dec;48(12):1462-1472.
33. Tiller D, Ittermann T, Greiser KH, Meisinger C, Agger C, Hofman A, Thuesen B, Linneberg A, Peeters R, Franco O, Heier M, Kluttig A, Werdan K, Stricker B, Schipf S, Markus M, Dörr M, Völzke H, Haerting J. [Association of Serum Thyrotropin with Anthropometric Markers of Obesity in the General Population.](https://www.ncbi.nlm.nih.gov/pubmed/27393002) Thyroid. 2016 Sep;26(9):1205-14.
34. Schunk M, Reitmeir P, Rückert-Eheberg IM, Tamayo T, Schipf S, Meisinger C, Peters A, Scheidt-Nave C, Ellert U, Hartwig S, Kluttig A, Völzke H, Holle R. Longitudinal change in health-related quality of life in people with prevalent and incident type 2 diabetes compared to diabetes-free controls. PLoS One. 2017 May 3;12(5):e0176895. doi: 10.1371/journal.pone.0176895. eCollection 2017.
35. Nolte IM, Munoz ML, Tragante, … Kluttig A… Snieder H, de Geus EJC. Genetic loci associated with heart rate variability and their effects on cardiac disease risk.Nat Commun. 2017 Jun 14;8:15805.
36. Wild PS, Felix JF…, Tiller D, … Zeller T, Vasan RS, Dörr M. Large-scale genome-wide analysis identifies genetic variants associated with cardiac structure and function. J Clin Invest. 2017 May 1;127(5):1798-1812.
37. Bächle C, Claessen H, Maier W, Tamayo T, Schunk M, Rückert-Eheberg IM, Holle R, Meisinger C, Moebus S, Jöckel KH, Schipf S, Völzke H, Hartwig S, Kluttig A, Kroll L, Linnenkamp U, Icks A. [Regional differences in antihyperglycemic medication are not explained by individual socioeconomic status, regional deprivation, and regional health care services. Observational results from the German DIAB-CORE consortium.](https://www.ncbi.nlm.nih.gov/pubmed/29370228) PLoS One. 2018 Jan 25;13(1)
38. Iqbal K, Dietrich S, Wittenbecher S, Krumsick J, Kühn T, Lacruz ME, Kluttig A , Prehn C, Adamski J, von Bergen M, Kaaks R, Schulze MB, Boeing H, Floegel A. Comparison of metabolite networks from four German population based studies. Int J Epidemiol. 2018 Dec 1;47(6):2070-2081.
39. Bahls M, Groß S, Baumeister SE, Völzke H, Gläser S, Ewert R, Markus MRP, Medenwald D, Kluttig A, Felix SB, Dörr M. Association of domain-specific physical activity and cardiorespiratory fitness with all-cause and cause-specific mortality in two population-based cohort studies. Sci Rep. 2018 Oct 30;8(1):16066.
40. Teumer A, Chaker L, … Greiser KH, … Meyer Zu Schwabedissen HE, … Tiller D, … Visser TJ, Medici M. Genome-wide analyses identify a role for SLC17A4 and AADAT in thyroid hormone regulation. Nat Commun. 2018 Oct 26;9(1):4455.
41. Ittermann T, Werner N, Lieb W, Merz B, Nöthlings U, Kluttig A, Tiller D, Greiser KH, Vogt S, Thorand B, Peters A, Völzke H, Dörr M, Schipf S, Markus MRP. Changes in fat mass and fat-free-mass are associated with incident hypertension in four population-based studies from Germany. Int J Cardiol. 2019 Jan 1;274:372-377.
42. Chak CM, Lacruz ME, Adam J, Brandmaier S, Covic M, Huang J, Meisinger C, Tiller D, Prehn C, Adamski J, Berger U, Gieger C, Peters A, Kluttig A, Wang-Sattler R. Ageing Investigation Using Two-Time-Point Metabolomics Data from KORA and CARLA Studies. Metabolites. 2019 Mar 5;9(3).
43. Wolf K, Bongaerts BWC, Schneider A, Huth C, Meisinger C, Peters A, Schneider A, Wittsiepe J, Schramm KW, Greiser KH, Hartwig S, Kluttig A, Rathmann W. Persistent organic pollutants and the incidence of type 2 diabetes in the CARLA and KORA cohort studies. Environment International 129 (2019) 221–228.
44. Kieback AG, Espinola-Klein C, Lamina C, Moebus S, Tiller D, Lorbeer R, Schulz A, Meisinger C, Medenwald D, Erbel R, Kluttig A, Wild PS, Kronenberg F, Kröger K, Ittermann T, Dörr M. One simple claudication question as first step in Peripheral Arterial Disease (PAD) screening: A meta-analysis of the association with reduced Ankle Brachial Index (ABI) in 27,945 subjects. PLoS One. 2019 Nov 4;14(11):e0224608.
45. Kartschmit N, Sutcliffe R, Sheldon MP, Moebus S, Greiser KH, Hartwig S, Thürkow D, Stentzel U, van den Berg N, Wolf K, Maier W, Peters A, Ahmed S, Köhnke C, Mikolajczyk R, Wienke A, Kluttig A, Rudge G. Walkability and its association with prevalent and incident diabetes among adults in different regions of Germany: results of pooled data from five German cohorts. BMC Endocr Disord. 2020 Jan 13;20(1):7. doi: 10.1186/s12902-019-0485-x.
46. Kartschmit N, Sutcliffe R, Sheldon MP, Moebus S, Greiser KH, Hartwig S, Thürkow D, Stentzel U, van den Berg N, Wolf K, Maier W, Peters A, Ahmed S, Köhnke C, Mikolajczyk R, Wienke A, Kluttig A, Rudge G.Walkability and its association with walking/cycling and body mass index among adults in different regions of Germany: a cross-sectional analysis of pooled data from five German cohorts. BMJ Open. 2020 Apr 28;10(4):e033941
47. Mühlenbruch K, Menzel J, Dörr M, Ittermann T, Meisinger C, Peters A, Kluttig A, Medenwald D, Bergmann M, Boeing H, Schulze MB, Weikert C. Association of familial history of diabetes or myocardial infarction and stroke with risk of cardiovascular diseases in four German cohorts. Sci Rep. 2020 Sep 21;10(1):15373.
48. Ho, J., Moriarty, F., Manly, J., Larson, E., Evans, D., Rajan, K., Hudak, E., Hassan, L., Liu, E., Sato, N., Hasebe, N., Laurin, D., Carmichael, P. and Nation, D., 2021. Blood-Brain Barrier Crossing Renin-Angiotensin Drugs and Cognition in the Elderly: A Meta-Analysis. Hypertension 2021, online ahead of print. <https://doi.org/10.1161/HYPERTENSIONAHA.121.17049>
